# Kinase Identification with Supervised Laplacian Regularized Least Squares

**DOI:** 10.1371/journal.pone.0139676

**Published:** 2015-10-08

**Authors:** Ao Li, Xiaoyi Xu, He Zhang, Minghui Wang

**Affiliations:** 1 School of Information Science and Technology, University of Science and Technology of China, 443 Huangshan Road, Hefei 230027, Anhui, China; 2 Centers for Biomedical Engineering, University of Science and Technology of China, 443 Huangshan Road, Hefei 230027, Anhui, China; International Centre for Genetic Engineering and Biotechnology (ICGEB), INDIA

## Abstract

Phosphorylation is catalyzed by protein kinases and is irreplaceable in regulating biological processes. Identification of phosphorylation sites with their corresponding kinases contributes to the understanding of molecular mechanisms. Mass spectrometry analysis of phosphor-proteomes generates a large number of phosphorylated sites. However, experimental methods are costly and time-consuming, and most phosphorylation sites determined by experimental methods lack kinase information. Therefore, computational methods are urgently needed to address the kinase identification problem. To this end, we propose a new kernel-based machine learning method called Supervised Laplacian Regularized Least Squares (SLapRLS), which adopts a new method to construct kernels based on the similarity matrix and minimizes both structure risk and overall inconsistency between labels and similarities. The results predicted using both Phospho.ELM and an additional independent test dataset indicate that SLapRLS can more effectively identify kinases compared to other existing algorithms.

## Introduction

Protein phosphorylation is one of the most pervasive posttranslational modifications and plays an important role in regulating nearly all types of cellular processes in organisms, including signal transduction, translation and transcription [1,2,3,4,5]. Phosphorylation is catalyzed by protein kinases [6], which regulate most cellular processes. More than one-third of proteins can be phosphorylated, and half of the protein kinases have intimate relationships with cancer and diseases [7]. Each protein kinase specifically catalyzes a certain subset of substrates, and deficiencies in protein kinases often cause diseases and cancers [6]. In this regard, identifying potential phosphorylation sites and their corresponding protein kinases is beneficial for elucidating molecular mechanisms.

Conventional experimental methods such as high-throughput biological technique mass spectrometry [8] were developed to identify phosphorylation sites. Although these experimental methods provide the foundation for understanding the mechanisms underlying phosphorylation, they are often costly and time-consuming. Additionally, although mass spectrometry methods can generate a large number of phosphorylated sites, most of these sites lack kinase information and the kinase that catalyzes the site is unknown. For example, Phospho.ELM, which is a verified phosphorylation site database, contains 37,145 phosphorylation sites, but only a small number of them (3,599 items) have corresponding kinase information. Due to the limitation of experimental methods, computational methods are required to identify protein kinases for specific phosphorylation sites based on data verified by experimental methods.

Currently, many computational methods have been developed for protein phosphorylation prediction. The first computational method proposed by Blom [9] was based on an artificial neural network algorithm using peptide sequences. Since then, a large number of methods have been developed, such as PPSP [10] and Musite [11]. PPSP adopts the Bayesian decision theory (BDT), which is based on an assumption that all flanking residues are independent of each other, to construct a classifier. Musite calculates the distance between two peptide sequences using the distance calculator Blosum62, which is a matrix reflecting the relationship between amino acids, and then constructs the classifier with support vector machine (SVM). Due to the increasing demand for kinase identification, a few machine learning-based methods have been developed in recent years. Among them, NetworKIN [12] uses consensus sequence motifs and a probabilistic protein association network. IGPS [13] is based on peptide sequence similarity and uses protein-protein interaction (PPI) information to control the false positive rate.

Despite the success achieved by these computational approaches, most of them neglect the geometry of the probability distribution [14], thereby hampering the improvement of prediction accuracy. For example, SVM only focuses on structural risk minimization and the quadrature encoder and thus ignores the intrinsic relationship between different amino acids. Additionally, the distance of two peptide sequences defined in Musite may fail to fulfill the triangle inequality [11]. To solve these problems, Belkin et al. [14] proposed a framework exploiting the geometry of the probability distribution; the test results showed that the proposed framework efficiently addressed the classification problems.

In this study, we propose a kernel-based supervised learning algorithm called Supervised Laplacian Regularized Least Squares (SLapRLS), which incorporates a new kernel construction method and brings together the spectral graph theory, regularization and the geometry of the probability distribution for kinase identification. In SLapRLS, reasonable translations are performed on a similarity matrix to force it to act as a kernel matrix [15]. Additionally, we introduce the overall inconsistencies between sample similarities and labels for each class [16] and minimize both the inconsistency and the structure risk. To compare the proposed algorithm with existing algorithms, we perform a 10-fold cross-validation using data retrieved from Phospho.ELM and compare SLapRLS with three classical algorithms: SVM, BDT and the k-nearest neighbor (KNN). To confirm the effectiveness and superiority of SLapRLS, an additional independent test dataset is used to compare SLapRLS and two other kinase identification tools: iGPS and NetworKIN. The results show that SLapRLS is more effective than the competitive algorithms and that the kernel matrix construction method is useful for the identification of kinases corresponding to known phosphorylation sites.

## Materials and Methods

### Data description

In this work, we extracted 37,145 experimentally verified phosphorylation sites from humans, including 3,599 sites with corresponding kinase information, from the most recent version of Phospho.ELM [17]. Among the sites with kinase information, 2,398 unique phosphorylation sites with kinase information in 934 proteins are obtained after removing the duplicated data. To overcome the over-estimation aroused by homology bias and redundancy, we cluster the protein sequences using Blastclust with a threshold of 70%; only one representation of each cluster is reserved [18]. As a result, 2,289 sites in 889 proteins are employed for the analysis. There are 1,823 serine (S)/ threonine (T) phosphorylation sites and 446 tyrosine (Y) phosphorylation sites. For each kinase, the corresponding phosphorylation sites are treated as positive data, whereas sites phosphorylated by other kinases are treated as negative data. Several kinases that contain too few known phosphorylated substrates are excluded to achieve reliable results. Finally, 23 types of kinases are obtained for investigation after removing the kinases that contain less than 20 positive items.

Because iGPS and NetworKIN use data retrieved from the Phospho.ELM database for model training, the test dataset in this study at least partially includes the training dataset of these two methods. This factor would inevitably result in the overestimation of the prediction performance for iGPS and NetworKIN. To obtain a fair comparison result, an independent dataset is adopted in this work [19]. Similarly, protein kinases in the independent test dataset that contain less than 20 items are also excluded to ensure the reliability of the results. Finally, we select 6 kinases in the independent dataset: PKC alpha, Erk2, Erk1, P38a, SRC and SYK.

### Algorithm

In this work, we propose that SLapRLS brings together spectral graph theory, regularization and the geometry of the probability distribution based on the regularized least squares (RLS) theory [14]. Similar to SVM, RLS is engaged in minimizing the structure risk [20]. SLapRLS is proposed based on the manifold assumption that similar samples tend to have similar results, and thus samples with the same label are predicted to have similar results. Therefore, the overall inconsistency between labels and pairwise similarities in the same class should be minimized. SLapRLS aims to minimize both structure risk and the overall inconsistency between labels and pairwise similarities.

### Feature description

In this work, we take full advantage of sequence information in modeling. A 15 amino acid local sequence is used to represent a candidate phosphorylation site that has 7 amino acids upstream and downstream of the phosphorylation site (S, T or Y). Thus, a phosphorylation site can be denoted as *s* = (*s*(1), *s*(2),…, *s*(8),…, *s*(15)), where *s*(*i*) represents the amino acid at the *i*_th_ position and *s*(8) is the phosphorylation site.

### Structure risk minimization and RLS

Structure risk minimization aims to minimize VC confidence and the summation of the empirical risk on each subset [21]. The square of the difference between the true label and the predicted result is often used as the loss function when calculating the empirical risk [22]. The optimization problem of RLS is shown as:

$$\underset{}{\text{min}}\frac{1}{l}\overset{}{\underset{}{\Sigma_{i\;=\;1}^{l}{\left(y_{i}-f\left(x_{i}\right)\right)}^{2}+\gamma_{A}||f{||}_{K}^{2}}}$$ (1)

where *y*_*i*_ and *f*(*x*_*i*_) represent the true label and the predicted result of the *i*_th_ sample, respectively.

### Inconsistency between labels and pairwise similarities

A good predictor should predict similar data with similar results, and thus the overall inconsistency between labels and pairwise similarities should be minimized [16]. The inconsistency contains two parts: the first is the inconsistency in the positive dataset and the second is the inconsistency in the negative dataset. The overall inconsistency is minimized and shown as:

$$\underset{}{\text{min}}\frac{1}{{\text{p}}^{2}}\Sigma_{\text{i},\text{j }=\;1}^{\text{p}}{\left(\text{f}\left({\text{x}}_{\text{i}}\right)-\text{f}\left({\text{x}}_{\text{j}}\right)\right)}^{2}{\text{W}}_{\text{ij}}+\frac{1}{{\text{n}}^{2}}\Sigma_{\text{i},\text{j }=\text{ p}+1}^{\text{p}+\text{n}}{\left(\text{f}\left({\text{x}}_{\text{i}}\right)-\text{f}\left({\text{x}}_{\text{j}}\right)\right)}^{2}{\text{W}}_{\text{ij}}$$ (2)

where *p* and *n* represent the number of positive data and negative results, respectively, and *W*_*ij*_ is the similarity between samples *x*_*i*_ and *x*_*j*_.

### Supervised LapRLS

Supervised LapRLS is based on the principle that both inconsistency between labels and pairwise similarities and structural risk should be minimized. The optimization problem aims to solve Eqs (1) and (2), which can be represented as Eq (3). This is a multiple objective optimization problem, and thus a weight parameter *γ*_*I*_ is introduced to weight the two objects [23].

$$\underset{}{\text{min}}\frac{1}{l}\Sigma_{i\;=\;1}^{j}{\left(y_{i}-f\left(x_{i}\right)\right)}^{2}+\gamma_{A}∥f{∥}_{K}^{2}+\gamma_{I}\left(\frac{1}{p^{2}}\Sigma_{i,j\;=\;1}^{p}{\left(f\left(x_{i}\right)-f\left(x_{j}\right)\right)}^{2}W_{ij}+\frac{1}{n^{2}}\Sigma_{i,j\;=\;p+1}^{p+n}{\left(f\left(x_{i}\right)-f\left(x_{j}\right)\right)}^{2}W_{ij}\right)$$ (3)

Data imbalance is a common problem in bioinformatics, in which negative data often have larger numbers than positive data. However, few methods have been proposed to address this problem. In this paper, we assign different penalty coefficients to different samples [24]. Therefore, SLapRLS aims to solve the optimization problem as:

$$\underset{}{\text{min}}\frac{1}{l}\Sigma_{i\;=\;1}^{l}c_{i}{\left(y_{i}-f\left(x_{i}\right)\right)}^{2}+\gamma_{A}∥f{∥}_{K}^{2}+\gamma_{I}\left(\frac{1}{P^{2}}\Sigma_{i,j\;=\;1}^{p}{\left(f\left(x_{i}\right)-f\left(x_{j}\right)\right)}^{2}W_{ij}+\frac{1}{N^{2}}\Sigma_{i,j\;=\;p+1}^{p+n}{\left(f\left(x_{i}\right)-f\left(x_{j}\right)\right)}^{2}W_{ij}\right)$$ (4)

where parameter *c* = (*c*_*1*_, *c*_*2*,_… *c*_*i*_,*…*,*c*_*l*_) is the penalty coefficient and *c*_*i*_ represents the misclassification cost of the *i*_th_ data *x*_*i*_. The misclassification cost contains two parts: the cost of misclassifying the positive samples as negative and the cost of misclassifying the negative samples as positive. Assuming that the number of class A is larger than class B, the model tends to classify the test data as class A. If the penalty of each class is equivalent, more samples in class B may be predicted as the wrong class. To solve the problem of data imbalance, the class with a smaller number is assigned a large penalty, while the class with a larger number is assigned a small penalty. The penalty coefficients for positive and negative data are set to *n*/(*p* + *n*) and *p*/(*p* + *n)* according to the numbers of positive and negative results. The two tuning parameters *γ*_*A*_ and *γ*_*I*_ in Eq (4) were selected from grid research in the range of [10^−5^, 10^5^] via ten-fold cross-validation [25] [26]; the values of the selected *γ*_*A*_ and *γ*_*I*_ for each kinase are listed in S1 Table. Belkin *et al*. proved that optimization problems that share similar object functions to Eq (2) were all convex optimization problems [14] and thus shared the same form as the solutions shown in (5). By using *K* to represent the Kernel function, we can calculate f*(*x*) as follows:

$$f^{*}\left(x\right)\;=\;\Sigma_{i\;=\;1}^{l}\alpha_{i}K\left(x_{i},x\right)$$ (5)

By solving the convex optimization problem shown in Eq (4), we can achieve ***a*** as follows:

$$\alpha \;=\;{\left(K+\gamma_{A}lI+\gamma_{I}l\left(\frac{1}{p^{2}}C^{-1}L_{P}K_{P,P}+\frac{1}{n^{2}}C^{-1}L_{N}K_{N,N}\right)\right)}^{-1}Y$$ (6)

Here, ***K*** is the kernel matrix with a size of (*p* + *n*)×(*p* + *n*) and can be denoted as $\left[\begin{array}{cc} K_{P,P} & K_{P,N}\\ K_{N,P} & K_{N,N} \end{array}\right]$, ***K***_***A*,*B***_ is a kernel matrix between two datasets A and B, and *P* and *N* represent the positive dataset and negative dataset, respectively. ***L***_***P***_ is the graph Laplacian of all positive data, which is given by

$${\text{ L}}_{\text{P}}\;={\text{ D}}_{\text{P}}{}^{-\frac{1}{2}}\left({\text{D}}_{\text{P}}-{\text{W}}_{\text{P},\text{P}}\right){\text{D}}_{\text{P}}{}^{-\frac{1}{2}}$$ (7)

where the diagonal matrix ***D***_***P***_ is given by:

$${\text{DP}}_{\text{i},\text{i}}\;=\;\Sigma_{{\text{x}}_{\text{j}}\in \text{P}}{\text{W}}_{\text{i},\text{j}},{\text{x}}_{\text{i}}\in \text{P}\;$$ (8)

Similarly, ***L***_***N***_ is the graph Laplacian of all negative data, which is given by

$$\;L_{N}\;=\;D_{N}{}^{-\frac{1}{2}}\left(D_{N}-W_{N,N}\right)D_{N}{}^{-\frac{1}{2}}$$ (9)

where the diagonal matrix ***D***_*N*_ is given by:

$${\text{DN}}_{\text{i},\text{i}}\;=\;\Sigma_{{\text{x}}_{\text{j}}\in \text{N}}{\text{W}}_{\text{i},\text{j}},{\text{x}}_{\text{i}}\in \text{N}$$ (10)

In (6), ***C*** is a diagonal matrix given by ***C*** = *diag*(*c*_*1*_,*c*_*2*_…*c*_*l*_), ***Y*** = (*y*_*1*_,*y*_*2*_…*y*_*l*_), ***W***_***P*,*P***_ is the similarity matrix between data in the positive dataset and ***W***_***N*,*N***_ is the similarity matrix between samples in the negative datasets.

### Similarity among samples

Because SLapRLS is based on sample similarities, the method used to calculate the similarity can have a large impact. Blosum62 is a matrix that reflects the relationship among amino acids and has been proven to be efficient for calculating pairwise similarity [11]. Here, we assume Blosum62 as matrix B and use *a* and *b* to represent two amino acids. Then, the similarity *W*_*i*, *j*_ between two samples *s*_*i*_ and *s*_*j*_ can be calculated as follows:

$${\text{W}}_{\text{i},\text{ j}}\;=\;\Sigma_{\text{t }=\;1}^{\text{w}}\text{sim}\left({\text{s}}_{\text{i}}\left(\text{t}\right),{\text{s}}_{\text{j}}\left(\text{t}\right)\right)$$ (11)

where *w* is the window size of a local peptide sequence and is set to 15 in this study. *s*_*i*_(*t*) represents the amino acid located in the *t*_th_ position of *s*_*i*_. Because the similarity between samples should be non-negative, we normalize B using:

$$sim\left(a,b\right)\;=\;\frac{B\left(a,b\right)-\text{min}\left(B\right)}{\text{max}\left(B\right)-\text{min}\left(B\right)}$$ (12)

sim(a, b)>0,

Because *sim*(*s*_*i*, *sj*_) is non-negative, it is easy to come to the conclusion that *W*_*i*, *j*_ is also non-negative.

### Kernel matrix construction

**Fig 1:**
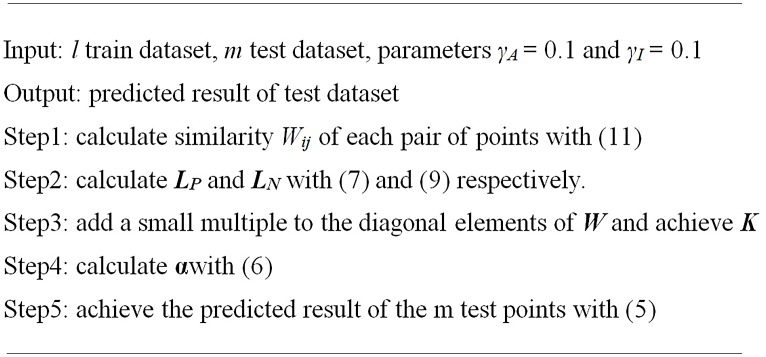
A summary of SLapRLS.

**Fig 2:**
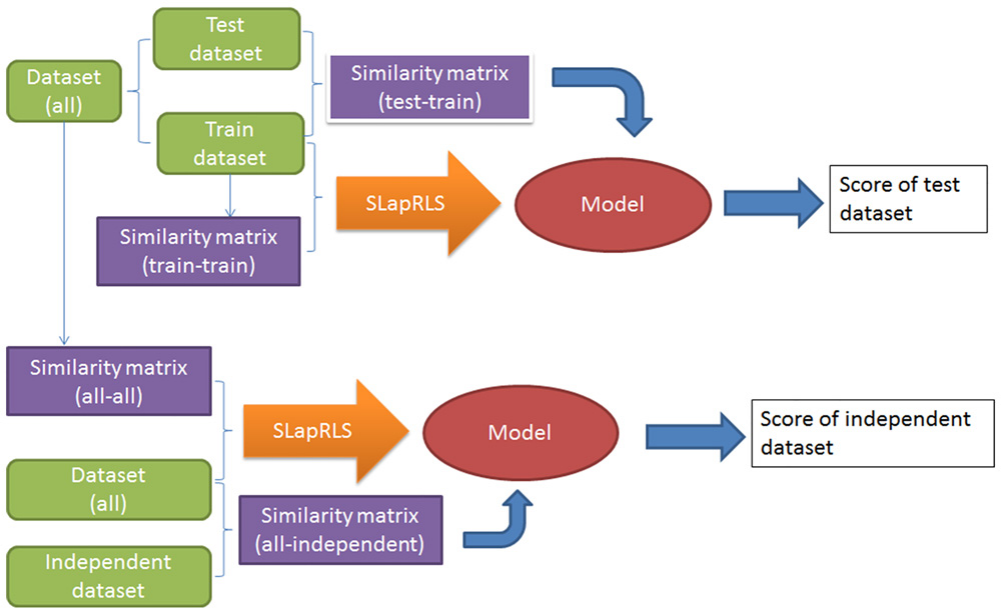
Procedure of this work. Firstly, label dataset are derived from Phospho.ELM, and it is split into train dataset and test dataset. Secondly, the model is developed using train dataset and its similarity matrix with SLapRLS, with which the predicted result of test dataset is achieved. Additionally, an independent test dataset is used. The model that predicts the independent dataset is developed with all the label dataset.

Kernel-based algorithms embed the dataset into a Hilbert space, and the kernel matrix completely reflects the relative positions of the samples in the embedding space. Several mathematically defined kernel functions exist (i.e., Gaussian kernel and spline kernel); these functions have been widely utilized in many research fields. However, these mathematically defined kernels often require few parameters and cannot effectively reflect the relationship between objects in a certain field. For example, in the field of kinase identification the Gaussian kernel needs the pairwise distance. A common way to calculate the distance is to encode the peptide sequence using quadrature encoding; then, the distance is calculated based on the Euclidean distance, which assumes that each amino acid is independent of the others. However, close relationships exist between amino acids, and thus calculating the similarity using the Gaussian kernel may miss important information from the substrate sequence [11]. Because a kernel function can reflect pairwise similarity, a more reliable way to calculate the similarity is to use expert knowledge and other information rather than the kernel function [27]. A kernel matrix should be symmetric and positive definite [28]. In this regard, we can perform translation on the similarity matrix to make it fulfill these two properties (symmetric and positive definite). The similarity matrix calculated with Eq (11) is symmetric, and thus we only need to add a small multiple to the diagonal elements of the similarity matrix to force it to be positive definite; then, the translated similarity matrix can be treated as the kernel matrix [15]. The summary of SLapRLS is shown in Fig 1, and the procedure of this work is shown in Fig 2.

### Performance evaluation

To evaluate the performance of the classifiers, we calculate the specificity (*Sp*), sensitivity (*Sn*), accuracy (*Acc*), precision (*Pre*) and Matthews correlation coefficient (*Mcc*). *Sp* and *Sn* represent the ratio of correctly predicted negative and positive sites, *Acc* indicates the percentage of truly predicted sites, and *Pre* indicates the ratio of true positive sites over predicted positive sites. *Mcc* reflects the balance quality between the true and predicted classes and illustrates the correlation between the true and predicted class. The definitions of *Sn*, *Sp*, *Acc*, *Pre* and *Mcc* are shown in Eqs (13), (14), (15), (16) and (17), respectively. The receiver operating characteristic (ROC) curve is widely used to evaluate the performance of a classifier in machine learning and plots (*1-Sp*, *Sn*) using each predicted value as the threshold. The corresponding area under the ROC curve (*AUC*) represents the overall accuracy of a classifier.

$$\text{Sn }=\;\frac{\text{TP}}{\text{TP}+\text{FN}}$$ (13)

$$\text{Sp }=\;\frac{\text{TP}}{\text{TN}+\text{FP}}$$ (14)

$$\text{Acc }=\;\frac{\text{TP}+\text{TN}}{\text{TP}+\text{TN}+\text{FP}+\text{FN}}$$ (15)

$$\text{Pre }=\;\frac{\text{TP}}{\text{TP}+\text{FP}}$$ (16)

$$\text{Mcc }=\;\frac{\text{TP}\times \text{FP}-\text{TN}\times \text{FN}}{\sqrt{\left(\text{TP}+\text{FN}\right)\times \left(\text{TP}+\text{FP}\right)\times \left(\text{TN}+\text{FN}\right)\times \left(\text{TN}+\text{FP}\right)}}$$ (17)

where *TP*, *FP*, *TN* and *FN* represent the number of true positives, false positives, true negatives and false negatives, respectively.

*AUC* is used as an overall performance measurement for comparison with other algorithms, and *Acc*, *Pre* and *Mcc* are utilized to evaluate the performance when the *Sp* is extremely high. Notably, we cannot use *AUC* as the evaluation for comparison with existing tools because the prediction scores are not available for these tools. In this situation, we make the comparison using the corresponding *Sn* value with a comparable *Sp* value by selecting a suitable threshold.

## Results and Discussion

### Comparison with other algorithms

We first compare our method with three existing algorithms (SVM, BDT and KNN) with a 10-fold cross validation using local peptide sequence information. When using SVM, peptide sequences are coded into numeric features using a quadrature encoder. In this study, we adopt the LIBSVM with an RBF kernel function [29], and parameters C andγin SVM are chosen by cross-validation. For BDT, the method introduced in PPSP [10] is adopted in this work. In KNN, the parameter K is set to 11 and the Blosum 62 matrix is employed to calculate the distance *d* among samples. Assuming *S*_*i+*_ is the total similarity between sample *s*_*i*_ and the top *K* nearest positive samples and *S*_*i-*_ is total similarity between sample *s*_*i*_ and the top *K* nearest negative samples, the final predicted score of sample *s*_*i*_ is denoted as the ratio of *S*_*i+*_ and *S*_*i-*_ [11].

**Fig 3:**
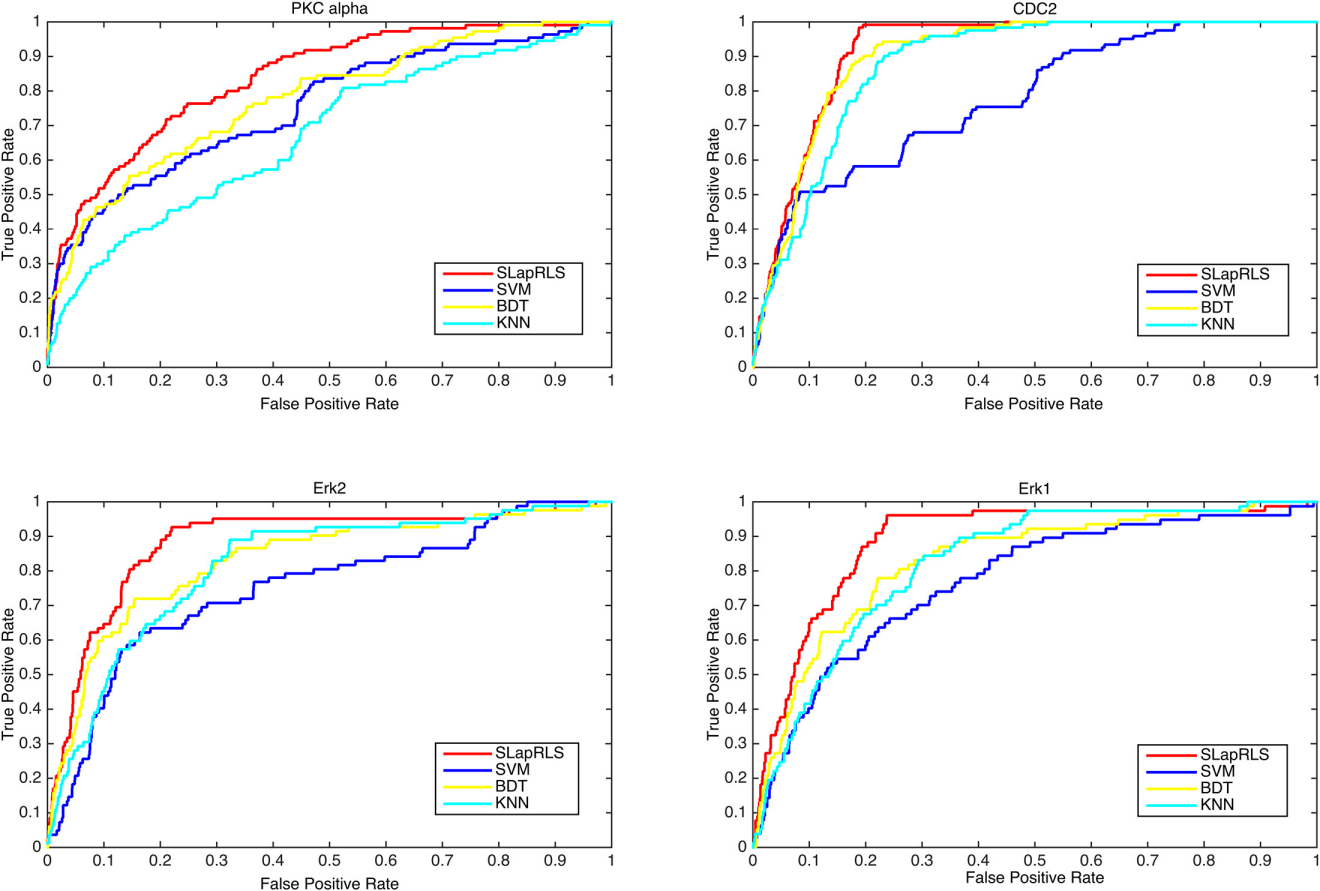
ROC curves of different algorithms. ROC curves of kinase Erk2, Erk1, CDC2 and PKC alpha achieved by four different algorithms are plotted. The red line, blue line, yellow line and cyan line represent SLapRLS, SVM, BDT and KNN, respectively.

The ROC curve is utilized to compare these four algorithms. The ROC curves for the Erk2, Erk1, CDC2 and PKC alpha kinases are shown in Fig 3, with the red line, blue line, yellow line and cyan line representing SLapRLS, SVM, BDT and KNN, respectively. As shown in Fig 3, the red line outperformed the other three lines, indicating that SLapRLS achieved better overall performance than the other algorithms.

To illustrate the robustness of our proposed method, we repeat the 10-fold cross validation five times and then compare the *AUCs*. Detailed results are listed in Table 1. As expected, SLapRLS achieves better performance than the other three algorithms on S/T/Y substrate kinases. For example, the average AUCs achieved by SLapRLS on kinase PKC alpha are 7.7%, 5.7% and 14.2% higher than SVM, BDT and KNN, respectively.

**Table 1:** Compared AUC values of the four algorithms: SLapRLS, SVM, BDT and KNN.

Methods	SLapRLS	SVM	BDT	KNN
S/T				
PKC alpha	**0.833LSBDT**	0.756LSBDT	0.776LSBDT	0.691LSBDT
ATM	**0.964LSBDT**	0.964LSBDT	0.905LSBDT	0.876L0.006
CDC2	**0.918L0.006**	0.714L0.006	0.894L0.006	0.880L0.006
Erk2	**0.882L0.006**	0.733L0.006	0.825L0.006	0.816L0.006
Erk1	**0.888L0.006**	0.760L0.006	0.831L0.006	0.815L0.006
AurA	**0.782L0.006**	0.652L0.006	0.724L0.006	0.718L0.006
BARK1	**0.747L0.006**	0.659L0.006	0.566±0.023	0.644±0.023
CaMK2a	**0.862a0.023**	0.682a0.023	0.688a0.023	0.757a0.023
CDK2	**0.887a0.023**	0.783a0.023	0.780a0.023	0.833a0.023
GSK3B	**0.806a0.023**	0.694a0.023	0.758a0.023	0.704a0.023
Ck2 a1ora2	**0.95420.023**	0.93220.023	0.93820.023	0.93020.023
MAPKAPK2	0.651PK2023	0.502PK2023	0.500PK2023	**0.716PK2023**
PDK1	**0.854PK2023**	0.787PK2023	0.811PK2023	0.842PK2023
P38a	**0.838PK2023**	0.708PK2023	0.781PK2023	0.732PK2023
PLK1	**0.734PK2023**	0.655PK2023	0.606PK2023	0.594PK2023
Y				
ABL1	0.560PK2023	**0.609PK2023**	0.467PK2023	0.519±0.018
EGFR	0.559±0.018	0.460±0.018	**0.594±0.018**	0.530±0.018
FYN	**0.730±0.018**	0.629±0.018	0.650±0.018	0.683±0.018
INSR	**0.547±0.018**	0.537±0.018	0.427±0.018	0.506±0.018
LCK	**0.638±0.018**	0.573±0.018	0.589±0.018	0.634±0.018
LYN	0.619±0.027	0.641 0.046	0.560±0.022	**0.672±0.022**
SRC	0.564±0.022	**0.570±0.022**	0.509±0.022	0.548±0.022
SYK	0.718±0.022	**0.726±0.022**	0.677±0.022	0.658±0.022

**Fig 4:**
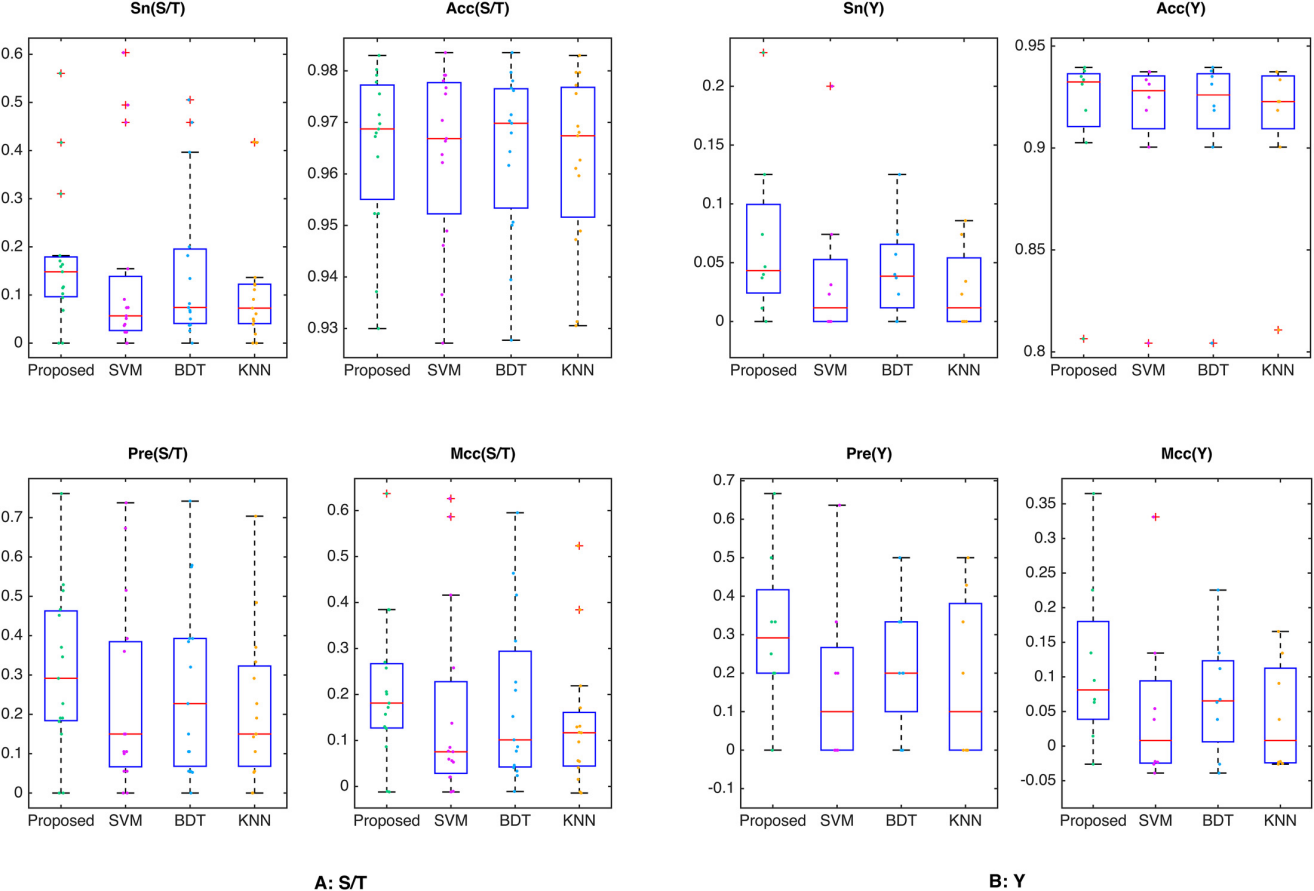
Comparison of the four algorithms at high stringency level (*sp = 0*.*99*). The *Sn*, *Sp*, *Acc* and *Mcc* values at high stringency level (*sp = 0*.*99*) of four algorithms on the S/T and Y kinases.

Additionally, *Sn*, *Acc*, *Pre* and *Mcc* are utilized to evaluate the performance of the four algorithms at a high stringency level (*Sp* = 0.99). The phosphorylated S/T and Y site kinases are divided into two groups (i.e., S/T substrate kinases and Y substrate kinases), and the performance of the two kinase groups are plotted in Fig 4. The results show that SLapRLS achieves higher *Sn*, *Pre*, *Acc* and *Mcc* and a slightly higher *Sp*. For example, the average *Sn* and *Pre* achieved by SLapRLS on Y substrate kinases are more than 2% and 9% higher than the other algorithms. Table 1 and Fig 4 show that SLapRLS also achieves better performance in S/T substrate kinases than Y substrate kinases.

**Fig 5:**
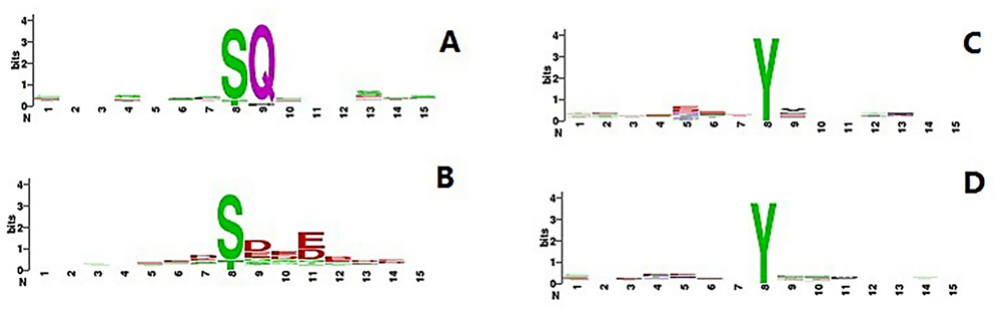
Weblogos of S/T substrate kinases and Y substrate kinases. A and C are the Weblogos of kinase ATM and ck2 alora2 and B and D are the Weblogos of kinase EGFR and INSR.

Because SLapRLS relies on the similarity between samples, a good similarity calculator is essential to achieve a satisfying performance. In this work, the similarity between samples is represented by peptide similarity, which is calculated using sequence information. The conservation is strong in the S/T substrate kinases but weak in the Y substrate kinases [30]. Fig 5 shows that the amino acid distributions of two S/T substrate kinases (ATM and ck2_alora2) have stronger conservation than two Y substrate kinases (INSR and EGFR). As shown in Fig 5, substrates of kinases with good performances tend to exhibit strong conservation, whereas the conservation of kinases with bad performances is weak. Therefore, local peptide sequence similarity may not effectively reflect the similarity between samples for kinases that have weak sequence conservation, and thus all four algorithms tend to achieve better performance for the S/T substrate kinases than the Y substrate kinases. It should also be noted that although only sequence information is used as an input feature, SLapRLS could actually address any type of data that can be used to calculate similarities between samples.

### Comparison with existing tools

To evaluate the performance of SLapRLS, we compare it with two existing kinase identification tools: iGPS and NetworKIN. Because cross validation is not available for iGPS and NetworKIN, we adopt an independent test dataset. We also compare *Sn* at the same high stringency *Sp* level using different algorithms. Comparison results are shown in Table 2. Although SLapRLS only uses sequence information as a feature while both iGPS and NetworKIN use protein-protein interaction information to filter the results, SLapRLS still achieves a satisfying performance (5 out of 6 kinases have a better performance compared with iGPS and NetworKIN). For instance, iGPS achieves an *Sp* of 0.780 and *Sn* of 0.525 for PKC alpha. To make a reasonable comparison with iGPS, we set the threshold to ensure that SLapRLS has a comparable *Sp* (0.782) and then calculate the corresponding *Sn* (0.983). The results show that the *Sn* of SLapRLS is 46% higher than *iGPS*. Thus, SLapRLS can effectively identify the corresponding kinase of the new site.

**Table 2:** Comparison among SLapRLS, iGPS and NetworKIN on independent test data.

Methods	iGPS		SLapRLS		NetworKIN		SLapRLS	
	*Sp*	*Sn*	*Sp*	*Sn*	*Sp*	*Sn*	*Sp*	*Sn*
PKC alpha	0.780	0.525	**0.782**	**0.983**	0.997	0.475	**0.997**	**0.559**
Erk2	0.466	0.709	**0.471**	**0.993**	0.865	0.278	**0.870**	**0.329**
Erk1	0.508	0.709	**0.510**	**0.974**	0.939	0.222	**0.942**	**0.247**
P38a	0.367	0.703	**0.369**	**0.865**	**1.000**	0.027	0.933	**0.054**
SRC	0.300	**0.875**	**0.310**	0.867	0.300	0.100	**0.310**	**0.867**
SYK	0.283	0.850	**0.301**	**0.850**	0.830	0.300	**0.849**	**0.400**

### A case study

To illustrate the usefulness of SLapRLS and to better elucidate the biological mechanism underlying phosphorylation, we perform an enrichment analysis on phosphorylation sites and their corresponding kinases. We adopt the kinase PKC alpha to illustrate the capability of SLapRLS to discover new phosphorylation sites. A total of 110 sites on 66 proteins phosphorylated by PKC alpha are extracted from the Phospho.ELM database. Additionally, we perform cross-validation on PKC alpha with SLapRLS and predict 56 candidate sites with the top 100 predicted scores. Enrichment analysis of the combined known and predicted proteins is performed using DAVID [31] to identify enriched pathways. As shown in Table 3, 6 KEGG pathways are highly enriched, with the most significant pathway related to the regulation of phosphorylation, and the corresponding Benjamin P-value is 9.2E-5. Additionally, 6 proteins in this pathway are predicted as substrates of PKC alpha by SLapRLS but are not included in Phospho.ELM, indicating that SLapRLS is able to identify potential corresponding kinases for known phosphorylation sites.

**Table 3:** Pathway enrichment analysis of PKC alpha.

Terms	Count	P-value	Benjamini P-values
Regulation of phosphorylation	14(6)	1.4e-7	9.2e-5
Cell migration	10(3)	4.2e-6	7.7e-4
Learning or memory	5(2)	1.7e-3	1.8e-2
Regulation of heart contraction	3(2)	2.4e-3	3.6e-2

## Conclusion

Phosphorylation plays an important role in multiple biological processes, and protein kinases have a tight relationship with many kinds of diseases. Thus, identifying the corresponding kinases for known phosphorylation sites is important. To overcome shortcomings such as costly and time-consuming experimental methods, the development of computational methods for kinase identification is urgently needed. At present, existing phosphorylation prediction-related computational methods neglect the geometry of the data distribution, and most kernel-based methods are based on distance. These distance-based methods often assume that the amino acids are independent when using local protein sequence information to calculate distances, while the connections between amino acids, which are also very important in expressing the relationships among samples, are neglected.

In this work, we propose the kernel-based algorithm SLapRLS that relies on similarity rather than distance and introduce the inconsistency between label and pairwise similarity to reflect the geometric distribution of the data. Instead of optimizing a single objective function, SLapRLS optimizes two functions: minimizing structure risk and the overall inconsistency between label and pairwise similarity. Because the kernel function reflects the closeness of two samples, we translate the kernel matrix from the similarity matrix instead of any famous kernel functions. The results show that SLapRLS outperforms three other algorithms (SVM, BDT and KNN) and two existing kinase identification tools (iGPS and NetworKIN).

It should to be noted that SLapRLS is based on a similarity matrix. Although only local sequence information is used as a feature in this work, SLapRLS is able to address any type of data as a feature, including characteristic types and numerical types. Although SLapRLS can solve kinase identification problems efficiently, there is also room for further improvement. For example, we only focus on local sequence information and disregard all other biological information. However, it has been proven that protein function and structural information is also useful for phosphorylation predictions [30, 32]. Therefore, such information could be utilized for kinase identification in a future work. To this end, a combination regulation should be introduced to incorporate different types of features.

## Supporting Information

S1 Table The selected parameters *γ*_*A*_ and *γ*_*I*_ for each kinase. (XLSX)
